# Protocol measuring horizontal gene transfer from algae to non-photosynthetic organisms

**DOI:** 10.1016/j.mex.2019.05.022

**Published:** 2019-06-25

**Authors:** James Weifu Lee

**Affiliations:** Department of Chemistry and Biochemistry, Old Dominion University, Norfolk, VA, 23529, USA

**Keywords:** Horizontal gene transfer measurement, Horizontal gene transfer, Biosafety risk assessment, Plasmid, Genomic transgenes, Cyanobacteria, *E. coli*, Genetically engineered blue-green algae

## Abstract

Horizontal gene transfer (HGT) is a natural process for an organism to transfer genetic material to another organism that is a completely different species, for example, from a blue-green alga to a non-photosynthetic bacterium. The phenomenon of HGT is not only of an interest to the science of molecular genetics and biology, but also to the biosafety issue of genetic engineering. The novel protocol reported here for the first time teaches how to measure HGT from a genetically engineered (GE) blue-green alga (gene donor) to wild-type *E. coli* (recipient). This novel protocol can be used to measure HGT frequency for both plasmid transgenes and/or genomic transgenes from a donor to recipient organism.

• According to this novel protocol, the HGT frequency may be calculated from the number of HGT recipient colonies observed, the number of recipient cells plated, and the donor-recipient co-incubation time.
• This approach can also help test the possible HGT routes to assess whether a HGT is through a direct cell-to-cell interaction or by an indirect cell-to-liquid environment-to-cell process.
• The protocol may be applied in full and/or in part with adjustments to measure HGT for a wide range of donor and recipient organisms of interest.

## Graphical abstract

**Figure:**
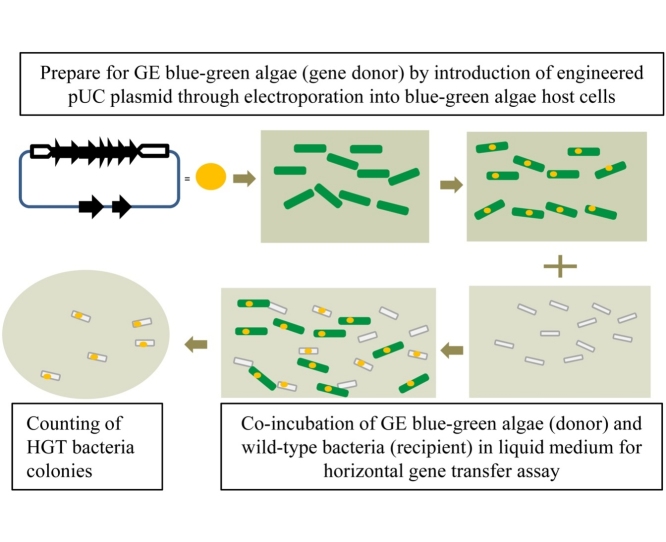

**Specifications Table**

**Table:** 

Subject Area:	• *Agricultural and Biological Sciences* • *Biochemistry, Genetics and Molecular Biology*
More specific subject area:	Horizontal gene transfer and biosafety risk assessment
Method name:	Horizontal gene transfer measurement
Name and reference of original method:	Experimental demonstration: T.H. Nguyen, C.L. Barnes, J.P. Agola, S. Sherazi, L.H. Greene, J.W. Lee, Demonstration of horizontal gene transfer from genetically engineered *Thermosynechococcus elongatus* BP1 to wild-type *E. coli* DH5α, *Gene*, **704** (2019) 49–58.
Resource availability:	*All resource information needed to reproduce this method is integrated in the paper (e.g., materials and reagent names, equipment, gene(s) donor and recipient organisms for horizontal gene transfer (HGT) assay, and the protocol for measuring horizontal gene transfer with HGT frequency equation).*

## Method details

### Materials and equipment

• Genetically engineered (GE) *Thermosynechococcus elongatus* BP1 or other blue-green algae (cyanobacteria) such as *Synechocystis* sp. strain PCC 6803 as a transgene(s) donor for horizontal gene transfer (HGT) assay.
• Wild-type *Escherichia coli* strain such as *E. coli* DH5α or the other non-photosynthetic bacteria to serve as a transgene(s) recipient for horizontal gene transfer (HGT) assay.
• BG-11 and BG-11_0_SA cyanobacteria culture media.
• Antibiotics (ampicillin, kanamycin, etc.).
• High-grade sterile LB agar plates with antibiotic and without antibiotic (control).
• Photosynthetic actinic light sources such as daylight fluorescent lamps.
• LI-COR LI-250A light meter.
• Properly autoclaved culture plates/tubes/flasks for cell growth.
• Temperature-controlled shaking incubators (IKA KS 4000 i control) for culture growth.
• Innova 2300 platform shaker.
• Olympus DP672 Microscope.
• Benchtop centrifuge (Eppendorf Centrifuge - 5418 R) for DNA preparation.
• Beckman Coulter Avanti J-26 Xp cooling centrifuge for cell harvesting and DNA preparation.
• Spectrophotometer for monitoring cell growth.
• Erlenmeyer flask of different size depending on the culture volume.
• Plasmid vectors such as pUC57-based pKB plasmid containing designer DNA constructs (transgenes) of interest including antibiotic selectable marker.
• BioRad C1000 thermal cycler for PCR.
• Thermoscientific nanodrop 2000 spectrophotometer for DNA quantification.
• UVP Benchtop UV transilluminator/PhotoDoc-it Imaging System.
• Gene pulser Xcell Electroporation BioRad (Total System).
• Thermoscientific Heratherm incubator IGS 100.
• Percival Environmental Control Growth Chamber/Incubator.
• Yamato Autoclave/ Sterilizer SM510.
• Class II biosafety cabinet (ESCO, Sentinel-Gold, Microprocessor Control System).
• Note: This list does not include any small generic laboratory equipment that are assumed to be available. Chemicals and other components can be used from any reliable company.
• Choice of appropriate antibiotics depends on the vector construct, bacteria such as *E. coli* and blue-green algae such as *Thermosynechococcus elongatus* BP1 hosts.
• Note: Several other vectors, *E. coli* and *Thermosynechococcus elongatus* BP1 strains are commercially available in the market; they can also be used for molecular cloning and horizontal gene transfer (HGT) assay as per the recommended protocol with appropriate selective antibiotics.

### Procedure

Protocol for measuring horizontal gene transfer

1 Prepare special plasmid containing designer transgene(s) of interest including an antibiotic selectable marker to create genetically engineered (GE) cyanobacteria to use as a transgene(s) donor for the horizontal gene transfer assay [1].
2 Create special plasmid-based and/or genomic-based GE cyanobacteria (such as *T. elongatus* BP1) through genetic transformation using electroporation followed by transformant colony selection and verification with PCR.
3 Photoautotrophically grow GE *T. elongatus* BP1 in BG-11 liquid culture medium.
4 Grow wild-type *E. coli* cells in liquid LB medium.
5 Harvest photoautotrophically grown GE *T. elongatus* BP1 cells from actively growing liquid culture (at mid logarithm-growth phase) by centrifugation using Beckman Coulter Avanti J-26 Xp centrifuge.
6 Harvest wild-type *E. coli* cells from actively growing liquid culture (at mid logarithm-growth phase) by centrifugation using Beckman Coulter Avanti J-26 Xp centrifuge.
7 Make cell suspension of GE *T. elongatus* BP1 in BG-11_0_SA liquid medium at the cell population density of about 10^7^ cells/mL.
8 Make cell resuspension of wild-type *E. coli* DH5α in LB liquid medium at the cell population density of about 10^7^ cells/mL.
9 Mix 15 mL of GE *T. elongatus* BP1 (donor) cells resuspension (in BG-11_0_SA) with 15 mL of wild-type *E. coli* DH5α (recipient) cells resuspension (in LB).
10 Incubate the two (donor and recipient) organisms at 1:1 population ratio (the “15 mL + 15 mL” mixture) on a shaker at 37 °C under continuous photosynthetic light intensity provided by daylight fluorescent lamps for a designated period of time ($t_{Incubation}$).
11 Take samples from liquid incubation co-culture and plate the liquid co-culture samples on LB agar medium in the presence and absence (control) of antibiotic; and spread (plate) a designated volume ($V_{Incubation}$), typically 100 μl, of the liquid co-culture (the donor-recipient co-incubation cell suspension liquid) onto the surface of a 45 mL antibiotic-containing LB agar medium per petri dish plate.
12 Incubate the LB plates at 37 °C for *E. coli* colony development and observation to count for HGT events for a period of 1, 2, 4, 7 and up to 14 days in combination with control experiments.
13 Count the HGT events by counting the number of *E. coli* DH5α (recipient) colonies ($N_{colony}$) on the antibiotic LB agar plates.
14 Verify the HGT *E. coli* colonies (cells) by observation using Olympus DP672 Microscope in combination with control experiments.
15 Verify the HGT *E. coli* colonies by colony PCR in combination with control experiments.
16 Calculate the horizontal gene transfer (HGT) frequency from the number ($N_{colony}$) of HGT recipient (*E. coli*) colonies observed per antibiotic LB agar selective plate, the volume ($V_{Incubation}$) of the donor-recipient co-incubation cell suspension liquid that was used in spreading onto the surface of an antibiotic-containing LB agar medium plate, the concentration ($C_{Recipient}$) of the recipient (*E. coli*) cells in the co-incubation liquid, and the donor-recipient co-incubation time ($t_{Incubation}$) according to the following HGT Frequency equation:

(1) $$HGT\;Frequency=\;\frac{N_{colony}}{V_{Incubation}\times C_{Recipient}\times t_{Incubation}}$$

## Method validation

### Materials and strains preparation

To measure HGT in accordance of this protocol, it is a preferred practice to use genetically engineered cyanobacteria (blue-green algae) with a selectable marker such as an antibiotic-resistant transgene as a transgene donor. Currently, genetically engineered (GE) cyanobacteria can be created through two types of genetic transformation: 1) plasmid-based genetic transformation and 2) integrative genetic transformation through homologous recombination into the host genomic DNA. In a plasmid transformant, the transgenes such as an antibiotic-resistant transgene are in a self-replicable plasmid like pUC57 that can somehow replicate also in certain host cyanobacterial cell. The copy number of the plasmid per cell may vary depending on how well the plasmid could replicate in the cyanobacterial cell. During cyanobacterial cell division, the distribution of plasmid to the daughter cells may be random in nature. Consequently, plasmid transformants tend to be less stable than the genomic transformants whose transgenes are integrated into their genomic DNA. It is likely that the transgenes carried by a plasmid may be more mobile for HTG than those integrated into the cyanobacterial genomic DNA. The protocol may be used to test this feature by measuring HGT frequency for both the plasmid transgenes and/or genomic transgenes from GE cyanobacteria to *E. coli*. Examples of materials and strains preparation including plasmid preparation and genetic transformation of cyanobacteria such as *Thermosynechococcus elongatus* BP1 (BA000039.2) for creation of GE cyanobacteria including GE *T. elongatus* BP1 (pKB plasmid transformant) has been experimentally demonstrated in the Lee Laboratory at Old Dominion University and recently reported in Ref. [1].

### Methods for measuring horizontal gene transfer

#### Horizontal gene transfer assay

**Fig. 1:**
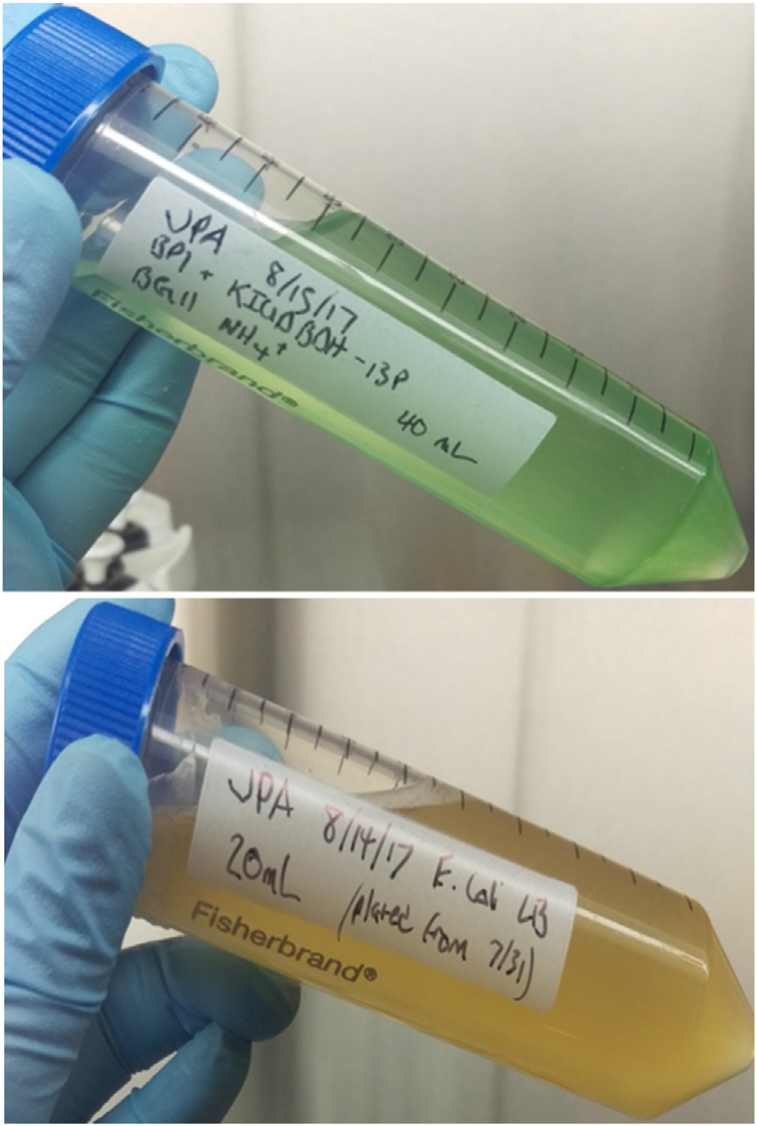
GE *T. elongatus* BP1 liquid culture cells suspension (Top) and wild-type *E. coli* cells suspension (bottom), both at a population density of about 10^7^ cells/mL and ready for use to co-incubate for the HGT assay [1].

GE *T. elongatus* BP1 (pKB plasmid transformant) cells were photoautotrophically grown at 45 °C in a Percival growth chamber in BG-11_0_SA medium containing kanamycin (40 μg/ml), and wild-type *E. coli* DH5α cells were grown in LB broth at 37 °C in a shaking incubator. The photoautotrophically grown cells of GE *T. elongatus* BP1 were collected by centrifugation. The supernatant was discarded and the cells were resuspended in fresh BG-11_0_SA medium without antibiotic. Both GE *T. elongatus* BP1 cells and wild-type *E. coli* cells were resuspended to population density of about 10^7^ cells/mL (Fig. 1).

**Fig. 2:**
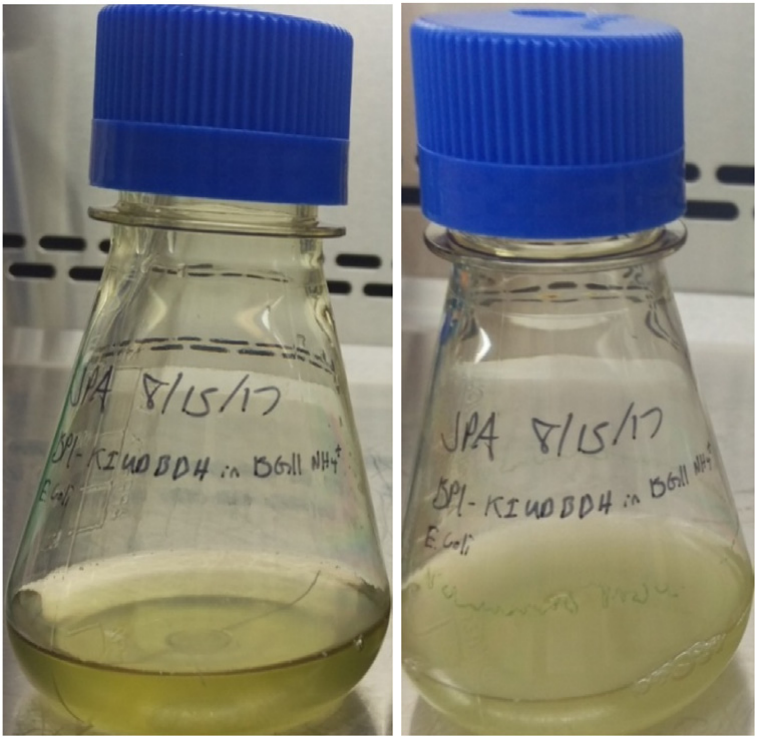
An example of the “15 mL + 15 mL” GE *T. elongatus* and wild-type *E. coli* cells liquid suspension co-incubation in a culture flask photographed at day 0 (left) and day 1 (right) for the HGT assay [1].

**Fig. 3:**
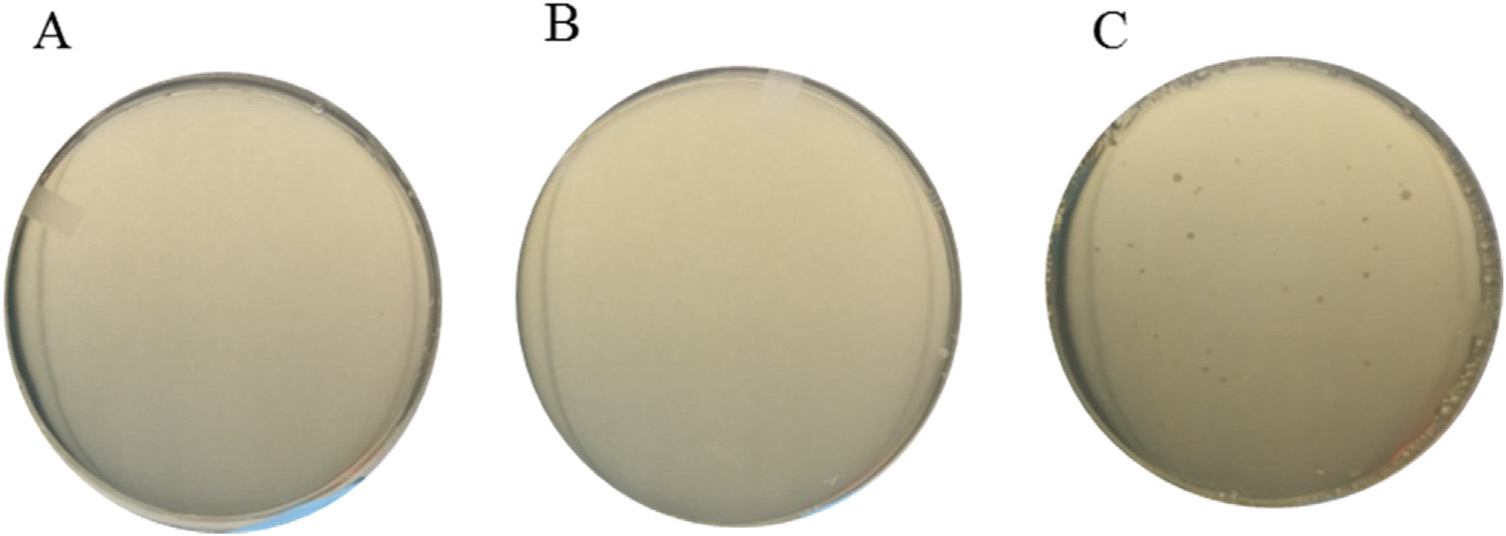
Examples of measuring HGT events by detection of *E. coli* growth and colony formation on LB agar plates. (A) No *E. coli* colonies were observed on a LB agar plate containing kanamycin 40 μg/mL from control 1. (B) No *E. coli* colonies were observed on a LB agar plate containing kanamycin 40 μg/mL from control 2. (C) *E. coli* colonies observed on a LB agar plate containing kanamycin 40 μg/mL after co-culturing with *T. elongatus* BP1 transformants carrying pKB plasmids. Co-cultures were grown for 2 days and additional 48 h of incubation on the plates.

Adapted from Nguyen et al. 2019 *Gene*, 704:49–58 [1].

Using the cells resuspension at a concentration of 10^7^ cells/mL, 15 mL cells resuspension of GE *T. elongatus* BP1 in BG-11_0_SA were mixed with 15 mL cells resuspension of wild-type *E. coli* DH5α in LB as shown in Fig. 2. The “15 mL + 15 mL” GE *T. elongatus* and wild-type *E. coli* liquid co-culture was incubated in a shaker at 37 °C under continuous photosynthetic light intensity of about 8 μE m^–2^ s^–1^ provided by daylight fluorescent lamps. In addition to the liquid incubation co-cultures, two controls were also set up using the *T. elongatus* BP1 transformant and wild-type *E. coli* DH5α. Control 1 consisted of 15 mL of *T. elongatus* BP1 transformant (10^7^ cells/mL) and 15 mL of LB broth without wild-type *E. coli* DH5α cells. Control 2 consisted of 15 mL of wild-type *E. coli* DH5α (10^7^ cells/mL) and 15 mL of BG-11_0_SA medium without *T. elongatus* BP1 transformant. These controls were also incubated in a shaking incubator at 37 °C under the same lighting conditions. Samples from each liquid co-culture and control were collected after 1, 2, and 3 days and were then sampled and spread on control LB agar plates and selection LB plates containing kanamycin. Typically, about 100 μl of liquid incubation co-culture sample was used to spread onto the surface of a 45 mL antibiotic-containing LB agar medium per petri dish (100 mm diameter, 15 mm deep) plate (Fig. 3). The LB plates were incubated at 37 °C under the same continuous photosynthetic lighting condition of 8 μE m^–2^ s^–1^. PCR was used to verify the presence of the transgene cassette DNA within these colonies [1].

As an example, to study whether GE *T. elongatus* BP1 (pKB plasmid transformant) has ability to transfer its pKB plasmid into wild-type *E. coli* DH5α, the two (donor and recipient) organisms were co-incubated at 1:1 population ratio for a designated period of time [1]. Then, the co-incubated liquid cell culture was sampled and plated on LB agar medium. The results for the control 1 (Fig. 3A), which only contained *T. elongatus* BP1 transformant, showed no colony growth on LB plates with and without kanamycin after 48 h of plate incubation at 37 °C under lighting. This indicated that *T. elongatus* BP1 could not grow on LB plates. Therefore, any colony formed on LB agar plates from this experiment would be *E. coli*. Bacterial growth was seen on LB plates without antibiotic for control 2 which contained wild-type *E. coli* only. However, control 2 did not have any colony growth on LB plates with kanamycin (Fig. 3B). The only colony growth observed on LB agar plates containing kanamycin was from co-cultured samples (the “15 mL + 15 mL” GE *T. elongatus* and wild-type *E. coli* liquid incubation co-cultures). Typically, the observed number of HGT *E. coli* colonies ranged from 20 to 75 per plate [1]. Because control 1 (Fig. 3A) eliminated the possibility of *T. elongatus* BP1 growing on LB agar plates, the colonies on LB plates with kanamycin were indeed *E. coli* (Fig. 3C). It is worth noting that these HGT *E. coli* colonies grew much slower on LB agar plates containing kanamycin compared to the control wild-type *E. coli* on plates without antibiotic which only took 24 h to form observable colonies instead of 48 h. Randomly selected HGT *E. coli* colonies on kanamycin plates were further analyzed by PCR to confirm the presence of the target plasmid within the HGT *E. coli* cells [1].

**Fig. 4:**
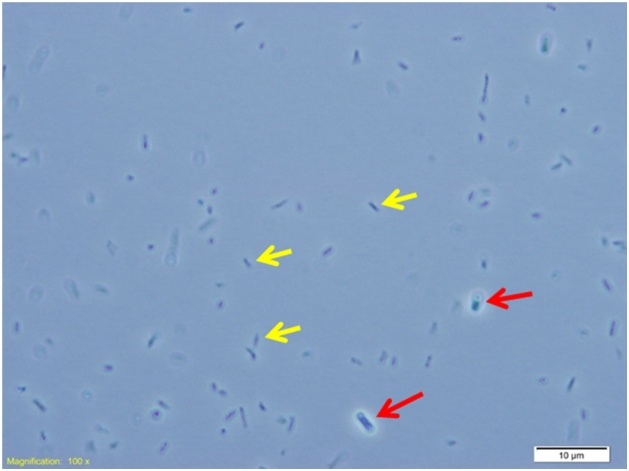
Example of visualizing the donor (*T. elongatus* BP1 transformants) and recipient (*E. coli*) cells in co-incubation liquid: microscopic image of liquid co-cultures containing *T. elongatus* BP1 transformants and *E. coli* DH5α after 2 days of co-incubation. Red arrows indicated *T. elongatus* BP1 transformants. Yellow arrows indicate *E. coli* DH5α.

Adapted from the Supplementary information of Nguyen et al. 2019 *Gene*, 704:49–58 [1].

**Fig. 5:**
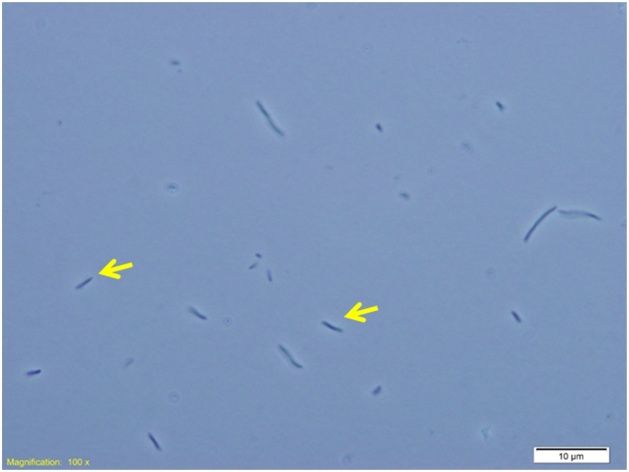
Example of examining the cells from selected HGT *E. coli* colonies: microscopic image of colony obtained from LB kanamycin plate of co-culture sample containing *T. elongatus* BP1 transformants and *E. coli* DH5α. Yellow arrows indicates *E. coli* DH5α cells.

Adapted from the Supplementary information of Nguyen et al. 2019 *Gene*, 704:49–58 [1].

The techniques of microscopic imaging can certainly be employed to visualize the donor (*T. elongatus* BP1 transformants) and recipient (*E. coli*) cells in the co-incubation liquid and to see the cells from selected HGT *E. coli* colonies. Fig. 4 presents a microscopic image of donor (*T. elongatus* BP1 transformants) and recipient (*E. coli* DH5α) cells in the co-incubation liquid that was examined under a light microscope (Olympus DP672 Microscope) after 2 days of co-incubation. Fig. 5 presents a microscopic image of the HGT *E. coli* DH5α cells from a colony obtained from LB kanamycin plate of co-culture sample containing *T. elongatus* BP1 transformants and *E. coli* DH5α. These microscopic examination results indicate that the protocol is working since the cells from the selected HGT recipient colonies were indeed the HGT *E. coli* DH5α cells as expected [1].

#### Calculating the horizontal gene transfer frequency from experimental data

Based on the protocol, the HGT frequency may be calculated from the number ($N_{colony}$) of HGT recipient (*E. coli*) colonies observed per antibiotic LB agar selective plate, the volume ($V_{Incubation}$) of the donor-recipient co-incubation cell suspension liquid that was used in spreading onto the surface of an antibiotic-containing LB agar medium plate, the concentration ($C_{Recipient}$) of the recipient (*E. coli*) cells in the co-incubation liquid, and the donor-recipient co-incubation time ($t_{Incubation}$) according to the HGT Frequency Eq. (1) described in the procedure above.

The HGT frequency expressed by this equation may be considered also as the probability for an HGT event to occur per recipient cell in a period of time under a given experimental condition.

Table 1 presents an example of using the Eq. (1) to calculate HGT frequency from the experimentally observed numbers of HGT recipient (*E. coli*) colonies per antibiotic LB agar plate under the given experimental conditions including the plated co-incubation liquid cell suspension volume of 100 μl, recipient cell concentration of 1/2 × 10^7^ *E. coli* cells /1000 μl in the co-incubation liquid, and the donor-recipient co-incubation time of 2 days. The HGT frequency (probability) from the plasmid transformants of cyanobacterium *Thermosynechococcus elongatus* BP1 (donor) to wild-type *E. coli* (recipient) was calculated to be in a range from 2.0 × 10^−5^ to 7.5 10^−5^ per cell day. That is, there could be about 20 to 75 HGT events per million recipient (*E. coli*) cells daily from the plasmid-based GE blue-green alga *Thermosynechococcus* under the given experimental conditions.

**Table 1:** Example of HGT frequency calculation with Eq. (1) using the HGT experimental data from the plasmid-based GE *Thermosynechococcus* (donor) to wild-type *E. coli* (recipient).

$N_{colony}$	20–75 HGT colonies per antibiotic LB plate
$V_{Incubation}$	100 μl per antibiotic LB plate
$C_{Recipent}$	1/2 × 10^7^*E. coli* cells/1000 μl
$t_{Incubation}$	2 days
HGT Frequncy	2.0 × 10^−5^–7.5 10^−5^ per cell day

As a conclusion, the HGT frequency can be calculated from the number of HGT recipient colonies observed, the number of recipient cells plated, and the donor-recipient co-incubation time. This approach can also help test for the possible HGT routes to assess whether a HGT is through a direct cell-to-cell interaction or by an indirect cell-to-liquid environment-to-cell process. The protocol may be applied in full and/or in part with adjustments to measure HGT for a wide range of donor and recipient organisms of interest, for example, from algae to non-photosynthetic organisms.

### “Tricks” and notes

1 The inability for certain cyanobacteria such as *Thermosynechococcus elongatus* BP1 to grow on LB agar plate makes the counting of HGT *E coli* colonies quite convenient. However, the protocol may be used for all cyanobacteria including those that can grow on LB agar plate as well, since cyanobacterial colonies typically have a blue-green color readily distinguishable from *E coli* colonies.
2 It is important to properly select the HGT co-incubation liquid medium, which should be able to accommodate both the donor (cyanobacteria) and recipient (*E. coli*) cells. Sometimes, it may be challenging to find a liquid culture medium that could accommodate both the donor (cyanobacteria) and recipient (*E. coli*) cells. A logical solution may be a 50–50 mixture of the donor liquid culture medium (such as BG-11_0_SA) and the recipient liquid culture medium (such as LB) as used in the present experiment.
3 The liquid co-incubation of the donor (cyanobacteria) and recipient (*E. coli*) cells should be performed under proper experimental conditions including not only the temperature but also the lighting conditions to accommodate both the donor and recipient cells in consideration of a natural environment such as in case of GE cyanobacteria accidently released into a natural pond environment, where they may meet with other bacteria like *E. coli.* The light intensity typically needs to be at least about 5 μE m^–2^ s^–1^ to ensure the blue-green algae being able to function properly with photosynthetic energy support.
4 After plating of HGT co-incubation liquid sample onto the surface of antibiotic LB agar selective medium, it is a preferred practice to incubate the antibiotic LB selective plates not only at a proper temperature (such as 37 °C) for HGT *E. coli* cells to grow, but also under a light intensity of at least about 5 μE m^–2^ s^–1^ to ensure the chlorophyll synthesis pathway in GE cyanobacteria is activated so that any potential cyanobacterial colony must have its green color that can be easily distinguished from an *E. coli* colony.
5 Note, the $C_{Recipient}$ used in the HGT frequency Eq. (1) described above is the cell population density (concentration) of the recipient (*E. coli*) in the co-incubation liquid, but not that of the donor (GE cyanobacteria). Therefore, in a “15 mL + 15 mL” GE *T. elongatus* and wild-type *E. coli* co-incubation liquid, the value for $C_{Recipient}$ was a half of the *E. coli* cell stock concentration (10^7^ *E. coli* cells/1000 μl) used in making the 50-50 co-culture of GE *T. elongatus* and wild-type *E. coli*.
6 Based on the protocol, the donor of genetic material for HGT assays does not have to be GE cyanobacteria. It can be a plasmid material such as the pKB plasmid tested in the experiment [1]. It can also be other sources of genetic materials such as DNA materials from certain dead organisms of interest. Therefore, it is practically appropriate to express the HGT frequency (probability) on the recipient basis as shown in Eq. (1) using $N_{colony}$ over $C_{Recipient}$, $V_{Incubation}$ and $t_{Incubation}$.
7 The protocol may be used to also test for certain possible routes of HGT such as to answer the questions of: 1) Does a HGT require a direct cell-to-cell interaction? or 2) Could a recipient cell pick up a piece of DNA in a liquid environment such as a plasmid that may be released from a donor cell (an indirect cell-to-liquid environment-to-cell process for HGT)? This type of questions can be answered by comparative HGT assays in presence and absence of GE cells and/or free plasmid DNA in incubation liquid as demonstrated in the experiments of Fig. 3 using the protocol.
8 Based on the present experimental result [1], the HGT frequency (probability) from the plasmid transformants of cyanobacterium *T. elongatus* BP1 to wild-type *E. coli* was estimated here to be in a range from 2.0 × 10^−5^ to 7.5 10^−5^ per cell day. The HGT probability from the genomic transformants of cyanobacteria to wild-type *E. coli* is likely to be rather low, which is yet to be measured. Special efforts such as increasing the incubation time and the donor and recipient cell population density for HGT liquid co-incubation with multiple replications may be needed to measure for such a potentially low probability genomic HGT event. This type of potentially challenging experiments with multiple replications may take months and even years of efforts to accomplish. It is a preferred practice to run a positive control experiment measuring HGT from a well-characterized plasmid transformant such as the pKB plasmid *T. elongatus* BP1 transformant to *E. coli* as a control in parallel with the long-duration main assay experiments to measure the genomic HGT event. This may help to ensure the long-duration experimental setups and conditions are all properly working so that the ultimate assay result whether positive or negative will represent the true outcome from the genomic HGT process of interest.
9 This protocol may be adjusted and applied in full and/or in part in combination with adjustments to measure HGT for a wide range of donor and recipient organisms from algae to non-photosynthetic organisms. For example, the recipient cell does not necessarily have to be *E. coli*. Other recipient organisms of interest such as yeast and human health-related bacteria including (but not limited to) *Shigella*, *Campylobacter*, and *Salmonella* may also be used for HGT assays in accordance of the protocol. Therefore, both the co-incubation liquid media and selective agar media plates may be adjusted in accordance of the protocol for the HGT assays based on the specific donor and recipient organisms of interest.

## Additional information

In contrast to the "vertical" transmission of DNA from the parent to its offspring of the same species, horizontal gene transfer (HGT) is a natural process for an organism to transfer genetic material to another organism that is not of the same species, for example, from a blue-green alga to a totally distinct non-photosynthetic bacterium. It is generally believed that HGT is used as a means of evolution [2]. The phenomenon of HGT sometimes is noticeable in certain genomic analysis. For example, there may be a piece of very different genomic DNA such as a distinct GC-rich DNA “island” comprising certain genes in otherwise a largely AT-rich genome of an organism. This type of distinct genetic material likely came from a very different (organism) species through a HGT event in the natural evolutionary process. Does such a HGT event occur at a time scale of million years, hundreds of years, or a few days? Currently, it is not entirely clear how often such a HGT event could happen between two different species. This paper outline a protocol to measure HGT from genetically engineered (GE) cyanobacteria which are also known as blue-green algae to non-photosynthetic organisms such as wild-type *E. coli.*

The phenomenon of HGT is not only of an interest to the modern science of molecular genetics and biology, but also to the biosafety issue of genetic engineering in relation to biofuel energy and environmental sustainability as well as public health and wellbeing. Synthetic biology using genetically engineered (GE) cyanobacteria has the potential to produce valuable products such as biofuels. In recent years, GE cyanobacteria have become a promising new agriculture avenue for photosynthetic renewable energy. This approach addresses concerns of fossil fuels including their contribution to the increase in greenhouse gases (such as CO_2_) in the Earth’s atmosphere and their inevitable depletion as the world’s major fuel source [[3], [4], [5], [6], [7], [8], [9], [10], [11]]. Photosynthetic production of various biofuels in GE cyanobacteria is accomplished by inserting a series of transgenes that will enable the direct conversion of photosynthetic metabolic intermediates to products such as ethanol and butanol [[12], [13], [14]]. Cyanobacteria are considered to be advantageous for several reasons which include the use of abundant raw materials (e.g. sunlight, water and CO_2_), decrease in competition for land and crops, and potential in alleviating the increase of CO_2_ in the atmosphere by being net carbon neutral [[15], [16], [17], [18]].

While the synthetic biology approach to photosynthetic biofuel production is promising, there are several key unresolved bio-safety questions that should be addressed before such methods are considered for wide commercial use. One of the main concerns is the ability of GE cyanobacteria to transfer their modified genes to other bacteria through HGT if they were to escape containment [19,20]. Through this process, it is possible that wild-type bacteria will be able to acquire designer transgenes such as those intended for biofuel production and/or antibiotic resistance. Because antibiotic resistance genes are widely used as a selective method for the genetic transformation of photosynthetic organisms and bacteria, HGT could increase the risk of proliferation which can have potential ramifications to human and animal health among other areas [21].

Better understanding the phenomenon of HGT between engineered and wild-type organisms (whether from the environment or common lab strains) will advance basic science knowledge in synthetic biology with microorganisms and could assist government agencies in the development of important policies and regulations for genetic engineering and use of GE cyanobacteria for biofuel production as well as other areas.

Therefore, this protocol is developed to determine whether a blue-green alga such as GE *Thermosynechococcus elongatus* BP1 has the potential to transfer its transgenes to wild-type *E. coli* and assess their possible HGT frequency. Earlier studies of HGT showed that *E. coli* can transfer genes to cyanobacteria [[22], [23], [24], [25], [26], [27]]. In the present work, the protocol of measuring horizontal gene transfer from GE blue-green algae to wild-type *E. coli* was experimentally demonstrated through the use of GE *T. elongatus* BP1 carrying a designer DNA construct with a plasmid to produce alcohol that was co-incubated with wild-type E. *coli* DH5α in a 1:1 ratio. HGT was then monitored on solid Luria Broth (LB) media with selective antibiotic for *E. coli* growth. The experimental results indicate that plasmids can be transferred from GE *T. elongatus* BP1 (transgene donor) to wild-type *E.coli* (recipient) after two days of liquid co-incubation [1]. The frequency (probability) of HGT for a plasmid transgene from the donor to the recipient organism was calculated, for the first time, from the experimental data based on the protocol.

In summary, this protocol could be extremely useful for testing of HGT between diverse microorganisms employing recombinant transgene DNA constructs. It utilizes the intrinsic differences in appearance and metabolism, with selectable antibiotic markers, to specifically screen for the transfer of gene(s) between transgenic blue-green algae and non-photosynthetic bacteria. Presence of the transgene(s) in the putative HGT recipients is then confirmed by PCR in combination of colony formation and re-streaking on culture plates with antibiotics. This type of assay may provide quantifiable information on possible unintentional genetic transfer from recombinant microalgae developed for commercial production to other microorganisms present in the environment. This approach could also be used to estimate horizontal gene transfer between *Escherichia coli* or other bacteria containing recombinant DNA transgenes and cyanobacteria (HGT in the opposite direction). Another possibility is to use an *E. coli* strain carrying a plasmid with a selectable marker different from that present in the cyanobacterial donor in this experimental scheme, then to subsequently screen for both selectable markers following the co-incubation step. Therefore, this novel protocol may be adjusted and applied in full and/or in part in combination with adjustments to measure HGT for a wide range of donor and recipient organisms from algae to non-photosynthetic organisms, which could be highly useful for a number of applications in various fields from the science of molecular genetics and biology to the biosafety of genetic engineering.

## Author’s contribution

The author JWL conceived the original idea of measuring horizontal gene transfer from algae to non-photosynthetic organisms and wrote this article for the novel protocol here with his original equation on HGT Frequency calculation. JWL first proposed the idea and wrote the method for measuring horizontal gene transfer in a research grant application in 2015. The proposed project was subsequently funded by the Biotechnology Risk Assessment Grant Program competitive grant award no. 2016-33522-25624 from the U.S. Department of Agriculture. This method has now been successfully practiced experimentally through the funded project work activity in measuring horizontal gene transfer. The graphic abstract was adapted and modified from Nguyen et al. 2019 *Gene*, 704:49–58 [1].
